# Cholangioscopy-guided retrieval of a migrated stent using a novel thin cholangioscope in a patient with Roux-en-Y gastrectomy

**DOI:** 10.1055/a-2780-0554

**Published:** 2026-02-09

**Authors:** Yuki Tanisaka, Shomei Ryozawa, Masafumi Mizuide, Akashi Fujita, Ryuichi Watanabe, Ryosuke Hamamura, Suguru Ito

**Affiliations:** 1 183786 Department of Gastroenterology, Saitama Medical University International Medical Center, Hidaka, Japan

**Fig. 1:**
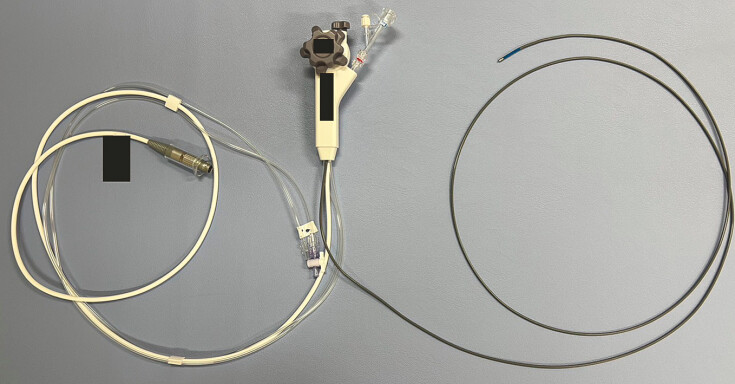
A thin cholangioscope (eyeMAX; Micro-Tech, China) measuring 219 cm in length, with a diameter of 9-Fr.

Stent migration can occur after endoscopic retrograde cholangiopancreatography (ERCP)-related procedures. Although various retrieval techniques have been reported 1 2 , removing a migrated stent remains technically challenging, particularly in patients with Roux-en-Y gastrectomy using a balloon enteroscope. Recently, a novel thin cholangioscope (eyeMAX; Micro-Tech, China), with a length of 219 cm and a diameter of 9-Fr ( Fig. 1 ), has enabled cholangioscopy-guided interventions to be performed under balloon enteroscopy 3 4 . We report a case of the successful cholangioscopy-guided retrieval of a migrated stent using a novel thin cholangioscope in a patient with Roux-en-Y gastrectomy.

**Fig. 2:**
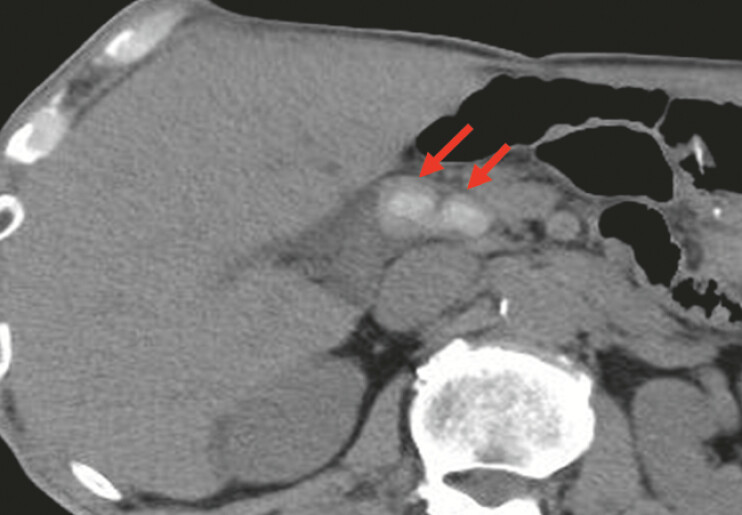
Computed tomography revealing multiple large stones in the common bile duct (red arrow).

**Fig. 3:**
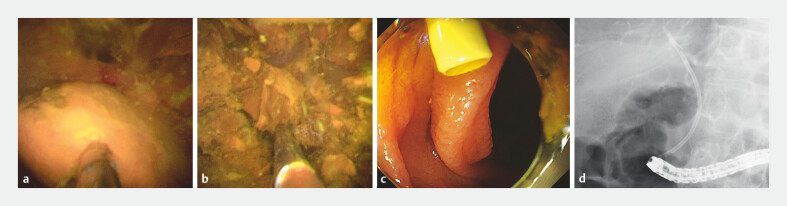
Findings of endoscopic retrograde cholangiopancreatography-related procedures. **a** and **b** Cholangioscopy revealing cholangioscopy-guided lithotripsy using a novel thin cholangioscope. Successful stone fragmentation was achieved. **c** and **d** Endoscopic and fluoroscopic findings revealing plastic stent placement due to the presence of residual stones.

**Fig. 4:**
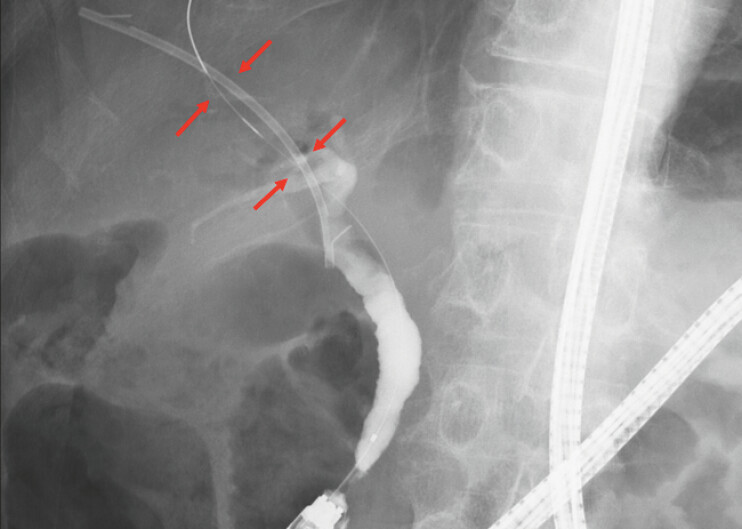
Cholangiography revealing the plastic stent migrated into the bile duct (red arrow).

**Fig. 5:**
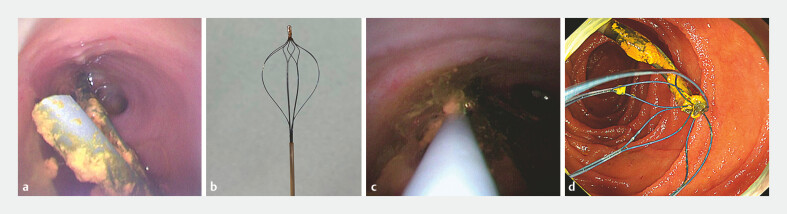
Cholangioscopic and endoscopic findings for stent retrieval. **a** Cholangioscopy revealing the migrated stent in the bile duct. **b** The retrieval basket (SpyGlass Retrieval Basket; Boston Scientific, USA), which can be inserted through the thin cholangioscope. **c** Cholangioscopy revealing the successful grasping of the stent flap. **d** Endoscopic findings revealing successful stent retrieval of a migrated stent.

A 74-year-old man with multiple large stones in the common bile duct, who had previously undergone Roux-en-Y gastrectomy, was referred to us ( Fig. 2 ). ERCP was performed using a short-type single-balloon enteroscopy (SIF-H290; Olympus Marketing, Japan) with a working length of 152 cm and a working channel of 3.2 mm in diameter 5 . Cholangioscopy-guided lithotripsy using a novel thin cholangioscope was performed, followed by the placement of a plastic stent due to the presence of residual stones ( Fig. 3 ). Two months later, residual stone extraction was attempted; however, upon reaching the papilla, the plastic stent was found to have migrated into the bile duct ( Fig. 4 ). Because fluoroscopy-guided stent retrieval using a basket catheter or a balloon catheter was unsuccessful, cholangioscopy-guided stent retrieval was attempted ( Video 1 ). The migrated stent was clearly visualized under cholangioscopy ( Fig. 5 **a** ). Therefore, we decided to capture the stent using a retrieval basket (SpyGlass Retrieval Basket; Boston Scientific, USA; Fig. 5 **b** ), which can be inserted through the thin cholangioscope. A distal flap of the stent was successfully grasped under direct cholangioscopic visualization ( Fig. 5 **c** ). Finally, a stent retrieval was achieved ( Fig. 5 **d** ).

Successful cholangioscopy-guided retrieval of a migrated stent using a novel thin cholangioscope in a patient with Roux-en-Y gastrectomy.Video 1

Since the retrieval of a migrated stent using a balloon enteroscope is technically challenging, cholangioscopy-guided retrieval with a novel thin cholangioscope is highly beneficial in such cases.

Endoscopy_UCTN_Code_TTT_1AR_2AL
